# HCV Tumor Promoting Effect Is Dependent on Host Genetic Background

**DOI:** 10.1371/journal.pone.0005025

**Published:** 2009-04-02

**Authors:** Naama Klopstock, Mark Katzenellenbogen, Orit Pappo, Miriam Sklair-Levy, Devorah Olam, Lina Mizrahi, Tamara Potikha, Eithan Galun, Daniel Goldenberg

**Affiliations:** 1 Goldyne Savad Institute of Gene Therapy, Hadassah-Hebrew University Medical Center, Jerusalem, Israel; 2 Bioinformatics and Microarray Unit, The Mina and Everard Goodman Faculty of Life Sciences, Bar-Ilan University, Ramat Gan, Israel; 3 Department of Pathology, Hadassah-Hebrew University Medical Center, Jerusalem, Israel; 4 Breast Imaging Center, Sheba Medical Center, Tel Hashomer, Israel; The University of Hong Kong, Hong Kong

## Abstract

**Background:**

The hepatitis C virus (HCV) is one of the major risk factors for the development of hepatocellular carcinoma (HCC). Nevertheless, transgenic mice which express the whole HCV polyprotein (HCV-Tg) do not develop HCC. Whereas chronic HCV infection causes inflammation in patients, in HCV-Tg mice, the host immune reaction against viral proteins is lacking. We aimed to test the role of HCV proteins in HCC development on the background of chronic inflammation *in vivo*.

**Methodology/Principal Findings:**

We crossed HCV-Tg mice that do not develop HCC with the Mdr2-knockout (Mdr2-KO) mice which develop inflammation-associated HCC, to generate Mdr2-KO/HCV-Tg mice. We studied the effect of the HCV transgene on tumor incidence, hepatocyte mitosis and apoptosis, and investigated the potential contributing factors for the generated phenotype by gene expression and protein analyses. The Mdr2-KO/HCV-Tg females from the N2 generation of this breeding (having 75% of the FVB/N genome and 25% of the C57BL/6 genome) produced significantly larger tumors in comparison with Mdr2-KO mice. In parallel, the Mdr2-KO/HCV-Tg females had an enhanced inflammatory gene expression signature. However, in the N7 generation (having 99.2% of the FVB/N genome and 0.8% of the C57BL/6 genome) there was no difference in tumor development between Mdr2-KO/HCV-Tg and Mdr2-KO animals of both sexes. The HCV transgene was similarly expressed in the livers of Mdr2-KO/HCV-Tg females of both generations, as revealed by detection of the HCV transcript and the core protein.

**Conclusion:**

These findings suggest that the HCV transgene accelerated inflammation-associated hepatocarcinogenesis in a host genetic background-dependent manner.

## Introduction

Hepatocellular carcinoma (HCC) is a significant health care burden worldwide, and chronic inflammation is a major risk factor for the development of HCC. The hepatitis C virus (HCV) is one of the major etiological HCC agents: it induces chronic liver inflammation and is responsible for the increased incidence of HCC in the developed world [1]. Although multiple cell culture models demonstrated oncogenic potential of several HCV proteins, most transgenic mouse models expressing these proteins did not develop HCC [2]. Furthermore, non infectious metabolic conditions causing chronic hepatitis also expose humans to an increased risk of developing HCC. Thus, determinants of HCC development on the background of chronic inflammation could be attributed to genetic/gender background, pathogen-derived or environmentally-dependent factors [3]–[5]. The aim of this study is to investigate the interplay between the factors contributing to the development of HCC in a mouse model expressing HCV proteins.

The previously generated HCV transgenic (HCV-Tg) mouse models produced inconsistent results concerning the role of HCV proteins in HCC development. Only one model that expressed the HCV core protein developed HCC with high incidence [6], and two models that expressed the HCV core together with E1 and E2 proteins developed HCC with very low incidence [7], [8]. However, other models containing similar transgenes, albeit produced in other laboratories, did not develop HCC [2]. Two groups created HCV-Tg mice expressing the whole HCV polyprotein [7], [9], however, none of these models are developing HCC (although in one model, HCC development was reported [7], this phenotype disappeared in the progeny throughout the next generations [10]). In transgenic animals, there is no inflammatory reaction against proteins expressed by a transgene, whereas in HCV-infected patients, the liver is inflamed. On the other hand, genetic models with chronic liver inflammation develop HCC at a high rate [11]–[13]. We and others have reported the importance of inflammatory mediators in HCC development [14]–[17].

In order to explore the hypothesis that HCV proteins may accelerate inflammation-associated liver carcinogenesis, and to identify and characterize the signaling pathways most likely to be affected, we crossed one of the existing HCV-Tg models, which produced detectable levels of HCV proteins [9], with the Mdr2 knockout (Mdr2-KO) mice, a model of inflammation-associated HCC. The Mdr2-KO mice develop liver tumors in about 100% of animals by the age of 16 months on the FVB/N genetic background; initially, this model was generated in the 129/OlaHsd strain, but due to a low fertility of the resulting mutants, the Mdr2-KO mutation was transferred into the FVB/N strain [11], [18]. We monitored HCC development in these hybrid mice by ultrasound, and, upon sacrifice, analyzed mitotic, apoptotic and gene expression patterns in their tumorous and non-tumorous liver tissues.

## Materials and Methods

### Mice

Mice were maintained at the Specific Pathogen-Free (SPF) unit, Faculty of Medicine, Hebrew University, under a 12 h light/dark cycle, and provided with food and water ad libitum. All animal experiments were performed according to national regulations and guidelines of the Institutional Animal Welfare Committee (NIH approval number OPRR-A01-5011). Founders of the FVB.129P2-Abcb4^tm1Bor^ (Mdr2-KO; old name FVB.129P2-P*gy*24^tm1Bor^) mice were purchased from the Jackson Laboratory (Bar Harbor, USA) [11]. Transgenic mice expressing the whole HCV 1b polyprotein were kindly provided by Prof. N. La Monica (IRBM, “P. Angeletti”, Pomezia, Italy) [9]. Double Mdr2-KO/HCV-Tg mice were produced by crossing between homozygous Mdr2-KO and HCV-Tg mice, and by backcrosses of the resulting F1 hybrid (both Mdr2- and HCV-heterozygous) with the parental Mdr2-KO strain. Appearance of tumors was monitored by ultrasound from the age of 10 months, using an ATL 5000 device (ATL, Bothell, WA) with linear transducer (12–15 mHz). Mice were sacrificed at the age of 14 months, when tumor appearance was detected in most Mdr2-KO mice.

### Genotyping

DNA from mouse tails or livers was purified by either the Wizard® Genomic DNA purification kit (Promega, Madison, WI) or by the Puregene® DNA isolation kit (Gentra systems, Minneapolis, MN). Genotype analysis was carried out by PCR analysis of DNA extracted from mouse tails using HCV transgene, Mdr2 and Neo primers (primers sequences are specified in the Table 1).

**Table 1:** PCR primers.

Gene	Sense primer	Antisense primer
HCV transgene	5′-ggcgcacatggcatcctc-3′	5′-gccaccgcaaggtctcgt-3′
Mdr2	5′-gctgagatggatcttga-3′	5′-tagccagatgatgg-3′
Neo	5′- aagaccgacctgtccg-3′	5′-tattcggcaagcaggcatcg-3′
Hprt	5′- gttaagcagtacagccccaaa-3′	5′- agggcatatccaacaacaaactt-3′
Gtf3c2	5′- ggtgtcacagtggctcaaga-3′	5′- aatggctgcttggagagaaa-3′
Sec24c	5′- tccacaacttggccctaaac-3′	5′- aaaccagctgccgaacatag-3′
Cyp4a31	5′- acaaggaccttcgtgctgag-3′	5′- cttgggacaggtgggtagag-3′
Erbb4	5′- atggccttccaacatgactc-3′	5′- cacctgccatcacattgttc-3′
Esm1	5′- gggatggaatgcaaagagac-3′	5′- agcgttcccttctccaatct-3′
Id3	5′- gaggagcttttgccactgac-3′	5′- tgaagagggctgggttaaga-3′
Ifrd1	5′- tccaagcctcctttcttgtg-3′	5′- gaccgctgctttctcttgtc-3′
Ndrg1	5′- tgtcccgagagctacatgac-3′	5′- ctcttgcaggagaccagtga-3′
Nrg1	5′- tcggtcagaacgaaacaaca-3	5′-tcgtggagtgatgagctgtg-3′
Ptgds	5′- aaccagtgtgagaccaagatca-3′	5′- ttggtgcctctgctgaatag-3′
Sox4	5′- ggcttcctaccttgcaacaa-3′	5′- tcgattgcagttcacgagag-3′
Styk1	5′- atggatggcctgctctatga-3′	5′- caggcccagatgacaaagtt-3′
Tff2	5′- cttggtgtttccacccactt-3′	5′- caccagggcacttcaaaga-3′
Ubd	5′- ggatttccaacccagctactc-3′	5′- gcagaaggatctggtcctgta-3′
Vnn1	5′- gggagacaagaagccgtgta-3′	5′- aacaggtgaagacgccaaac-3′

### Harvesting of mouse tissue

Non fasting mice were anesthetized with isoflurane and sacrificed by cervical dislocation. Livers were rapidly excised and weighed; a part of the liver was fixed with 4% buffered formaldehyde for histological analysis, and the remaining tissue was rapidly frozen in liquid nitrogen and stored at −80°C until further use.

### RNA extraction and analysis

Total RNA was isolated from snap frozen mouse liver tissues with the Trizol® reagent (Invitrogen, Carlsbad, CA) as described by the manufacturer. Whole genome gene expression profiling of RNA samples was performed using Affymetrix GeneChip® Mouse Exon 1.0 ST Array (Affymetrix, Santa Clara, CA).

### Calculation of gene expression values

The raw data were processed by the Partek® Genomics Suite™ (Partek GS) (Partek Inc., St. Louis, MO) software. RMA algorithm was applied for summarization, and the normalization was performed by the quantile method. The normalized expression values were processed by the Partek batch removing tool. The analysis was repeated twice: on “extended” and “core” subsets of probe-sets and when the probe-set mean was applied for evaluation of transcript cluster expression level. The processed data were submitted either to fold-change analysis with a threshold of 1.85, to hierarchical clustering with the Eisen software [19], or to SPIN analysis (sorting points into neighborhoods (sorting algorithm)) [20], which enables arrangement of expression profiles in the order of similarity (“side-to-side” sorting variant).

### Analysis of alternative splicing

The alternative splicing calculation was performed using the definition of splicing index as average log_2_ transformed relations between expression levels of individual probe-sets (representing individual exons) and transcript clusters (evaluation of gene expression level) [21]. Statistical significance of the differences between splicing indices in different experimental groups was evaluated with the ANOVA test for comparison between the non-matched samples or paired T-test for matched samples (tumors and non-tumorous tissues). The significant difference in the splicing index was considered to be a sign of change of the tested exon inclusion levels. Only probe-sets and transcripts with a maximal expression level >5 (in log_2_ scale) were used.

### Confirmation of differential gene expression by RT–PCR

Reverse transcription of total RNA was performed using the MMLV Reverse Transcriptase and random hexamer primers (Promega, Madison, WI). Semi-quantitative PCR was performed using Super-therm Taq Polymerase (JMR, UK). For each gene, the cDNA concentration and the number of PCR cycles were established in the linear amplification range. Expression levels for each gene were normalized against one of the following housekeeping genes: Hprt, Gtf3c2 or Sec24c (PCR primers are specified in the Table 1). Pictures of agarose gels were taken by B.I.S. 202D BioImaging System (Amersham Pharmacia Biotech., USA). The intensities of bands and p-values were obtained using the ImageGauge Ver. 4.0 program for quantification of images (Fujifilm Science Lab.). Real-time PCR was run in triplicates using the TaqMan Universal PCR Master Mix, primers, probe sets, and the ABI PRISM 7700 Sequence Detector System from Applied Biosystems (Foster City, CA). Threshold cycle numbers (Ct) were determined with the Sequence Detector Software (version 1.6; Applied Biosystems) and transformed using the ΔCt method as described by the manufacturer. The relative quantification values for each gene were normalized against the endogenous housekeeping gene Sec24c, which was one of the most uniformly expressed genes in all RNA samples subjected to global gene expression profiling. Relative quantitation value of each tumorous sample was compared to the relative quantitation value of its matched non-tumorous sample.

### Immunohistochemistry

Immunostaining was performed on four-micrometer-thick, formalin-fixed, paraffin-embedded liver tissue sections by standard procedures. Antigen retrieval was done with citrate buffer, pH 6.0 (HCV core, β-catenin, galectin-1, and Ubd/Fat10) or glycine buffer, pH 9.0 (cyclin D1) in a microwave. Primary antibodies were: anti-HCV-core Ab (ABR Affinity Bioreagents, Golden, CO), rabbit anti-cyclin D1 SP4 clone (Diagnostic Biosystems, Pleasanton, CA), anti-β-catenin (BD Bioscience, San Jose, CA), anti-galectin-1 and anti-Ubd/Fat10 (SC-19277 and SC-51086, respectively, both from Santa Cruz biotechnology, Santa Cruz, CA). We used the SuperPicture™-HRP polymer conjugate broad spectrum kit (Zymed, San Francisco, CA) as secondary antibody for HCV core and Cyclin D1, Envision®+HRP labeled polymer (DAKO, Carpinteria, CA) for β-catenin, LSAB®+System-HRP (Dako, Carpinteria, CA) for galectin-1, and HRP-conjugated donkey anti-goat antibody (Jackson ImmunoResearch Laboratories, West Grove, PA) for Ubd/Fat10 staining. Color development with diaminobenzidine was done using the Zymed kit as mentioned above. Scores for galectin-1 staining were assessed according to evaluation of average level of staining all along the section.

### Statistical analysis of the data

Significance of the difference in tumor incidence between mouse groups was tested by Chi-Square-test; significance of differences in the levels of blood enzymes, in the levels galectin-1 immunostaining, in the frequency of mitosis and apoptosis, and in the semi-quantitative RT-PCR was tested by t-test. Statistical evaluation of differential expression between the experimental groups was performed using ANOVA test.

## Results

### Effect of the HCV transgene on hepatocarcinogenesis in Mdr2-KO mice

**Figure 1:**
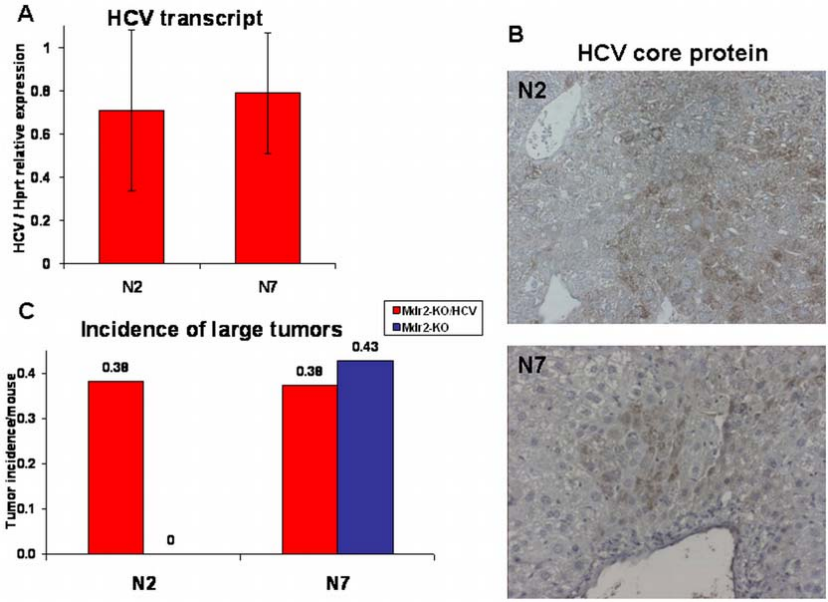
HCV transgene expression and incidence of large liver tumors in female mice from the N2 and N7 generations. (A) Similar expression levels of the HCV transcript in 14-month-old Mdr2-KO/HCV mice from the N2 and N7 generations. Average levels of the HCV transcript were determined by semi-quantitative RT-PCR relative to expression level of the housekeeping gene Hprt in 5 females from the N2 generation and in 15 females from the N7 generation. (B) Similar expression of the HCV core protein in 14-month-old Mdr2-KO/HCV mice from the N2 and N7 generations. Representative immunohistochemical staining against HCV core protein in females from the N2 and N7 generations (magnification: ×200). (C) Higher incidence of large liver tumors (diameter over 1 cm) per mouse in 14-month-old Mdr2-KO/HCV compared to Mdr2-KO female mice from the N2, but not from the N7 generations. Incidence of all tumors is shown in Table 2. Red bars – Mdr2-KO/HCV group, blue bar – Mdr2-KO group.

The first breeding between homozygous HCV-Tg and Mdr2-KO mice (having C57Bl/6 and FVB/N genetic backgrounds, respectively) produced a generation of HCV- and Mdr2-heterozygotes (Fig. S1). The second backcross of these mice to the maternal Mdr2-KO strain produced the N2 generation which contained 25% of the C57Bl/6 genetic background. We continued to produce backcrosses till the N7 generation, an incipient congenic strain containing only 0.8% of the donor C57Bl/6 genetic background and 99.2% of the recipient FVB/N genetic background. The produced hybrid mice were followed for HCC development, and comparison between the N2 and N7 generations was performed. Independently of the presence of the HCV transgene, Mdr2^+/−^ mice did not develop HCC upon sacrifice at 18 months of age, whereas all Mdr2-KO mice did produce liver tumors at 14 months of age (Table 2). Most tumors were well-differentiated HCC as determined by previously described criteria [18] and by immunostaining for reticulin fibers (not shown). Immunohistochemical analysis of liver tissues from HCV-Tg mice with antibodies to the core protein showed non-uniform distribution of HCV transgene expression both in Mdr2-KO and Mdr2-heterozygous mice. Levels of the HCV transgene expression were similar between Mdr2-KO/HCV-Tg mice from the N2 and N7 generations (Fig. 1, A and B). However, a significant difference in the incidence of large tumors was observed upon comparing N2 female mice with and without the HCV transgene: only N2 female mice with the HCV transgene had tumors with diameters over 1 cm (p = 0.0445; Table 2 and Fig. 1C). In contrast, in mice from the N7 generation, the incidence of large tumors was independent of the presence of the HCV transgene and was similar to that in Mdr2-KO/HCV-Tg mice from the N2 generation. The 25% of the C57Bl/6 genetic background present in the N2 generation had a tumor suppressive effect in the Mdr2-KO mice not having the HCV transgene (Fig. 1C). There was a statistically significant reduced incidence of tumors with a diameter over 1 cm between the animals from the N2 and N7 generations (Chi-Square-tests for both all mice (p = 0.0237) and for females (p = 0.0299); Table 2). The tumor promoting effect of the HCV transgene was statistically significant only on the partially suppressive N2 genetic background, and only in females (p = 0.0445).

**Table 2:** Incidence of HCC in 14-month-old Mdr2-KO/HCV-Tg and Mdr2-KO mice from the N2 and N7 generations.

Incidence of HCC in N2			Tumor size: >1.2 cm	Tumor size: 1–1.2 cm	Tumor size: 0.8–1 cm	Tumor size: 0.5–0.7 cm
genotype	sex	No. of mice				
**MDR2-KO/HCV**	m	16	1	1	1	5
	f	13	2	3	0	1
	all	29	3	4	1	6
**MDR2-KO**	m	10	0	1	0	3
	f	8	0	0	1	3
	all	18	0	1	1	6

Incidence of HCC in N7			Tumor size: >1.2 cm	Tumor size: 1–1.2 cm	Tumor size: 0.8–1 cm	Tumor size: 0.5–0.7 cm
genotype	sex	No. of mice				
**MDR2-KO/HCV**	m	13	0	1	2	4
	f	16	4	2	5	3
	all	29	4	3	7	7
**MDR2-KO**	m	12	2	1	0	5
	f	14	5	1	3	2
	all	26	7	2	3	7

**Figure 2:**
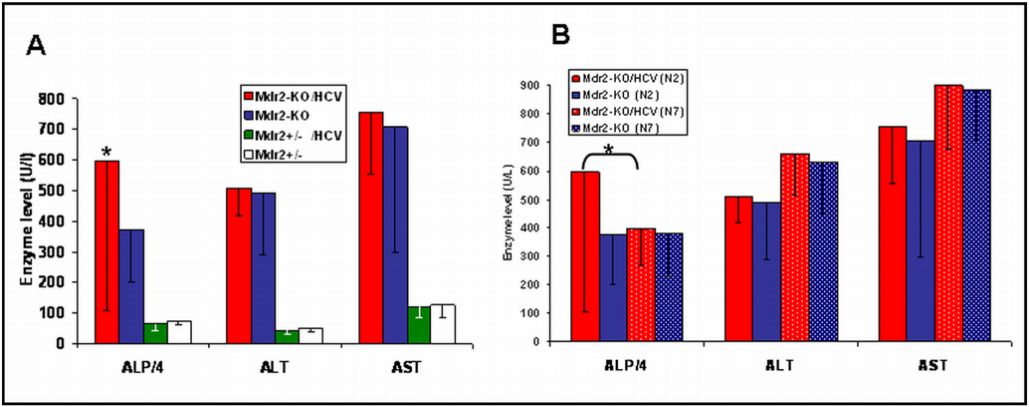
Levels of liver enzymes in the serum of 14-month-old Mdr2-KO female mice. Liver enzymes: Alkaline Phosphatase (ALP) Alanine aminotransferase (ALT), and Aspartate aminotransferase (AST) were measured in serum of 14 month old female mice. Graph represents average levels ±SD. (A) Comparison of enzymes levels (U/l) between Mdr2-KO and Mdr2^+/−^ with and without HCV-Tg from the N2 generation. (B) Comparison of enzymes levels (U/l) of Mdr2-KO/HCV and Mdr2-KO females from N2 and N7 generation. Statistically significant difference was found in ALP levels between Mdr2-KO/HCV females and Mdr2-KO females of the N2 generation only (* p = 0.04).

Higher levels of liver enzymes (alanine aminotransferase, aspartate aminotransferase and alkaline phosphatase) were found in the serum of all Mdr2-KO mice compared to Mdr2^+/−^ controls (Fig. 2A), as expected [15]. However, the only statistically significant difference between Mdr2-KO/HCV mice and Mdr2-KO mice from the N2 generation was in the level of alkaline phosphatase, found in females only (p = 0.04). Nevertheless, there was no direct correlation between the level of alkaline phosphatase and liver tumor load/incidence in individual Mdr2-KO/HCV mice. Similarly, high levels of serum liver enzymes were also measured in Mdr2-KO mice of the N7 generation; however, the level of alkaline phosphatase was not specifically increased in the Mdr2-KO/HCV-Tg females compared to Mdr2-KO females not expressing the HCV transgene (Fig. 2B).

### Effect of the HCV transgene on mitosis and apoptosis in Mdr2-KO mice

**Figure 3:**
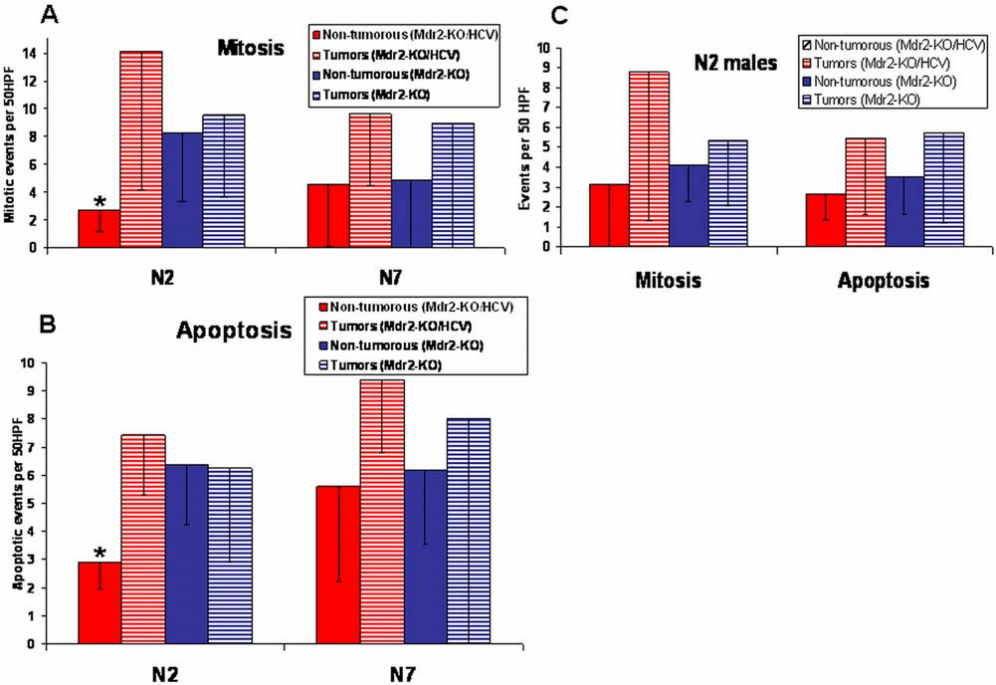
Mitotic and apoptotic events in the livers of mice from the N2 and N7 generations. Events were counted per 50 HPF ±SD in H&E stained liver sections of tumors (0.3–1 cm diameter) and non-tumorous tissue samples from 14-month-old mice. (A) Reduced mitotic events (p = 0.016) in non-tumorous liver tissues of Mdr2-KO/HCV females compared to Mdr2-KO female mice from the N2, but not from the N7 generations. (B) Reduced apoptotic events (p = 0.002) in non-tumorous liver tissues of Mdr2-KO/HCV females compared to Mdr2-KO female mice from the N2, but not from the N7 generations. (C) Similar intensities of mitotic and apoptotic events in the livers of male mice from the N2 generation. There was no statistically significant difference between groups.

To investigate the potential mechanisms for the effect of the HCV transgene on hepatocarcinogenesis, we compared mitotic and apoptotic events in non-tumorous and tumorous liver tissues of the Mdr2-KO and Mdr2-KO/HCV-Tg females from the N2 and N7 generations (Fig. 3A – mitosis, Fig. 3B - apoptosis) and of the males from the N2 generation (Fig. 3C). Interestingly, we observed a statistically significant decrease of mitosis (p<0.016) and apoptosis (p<0.002) in the non-tumorous tissues of the Mdr2-KO/HCV-Tg females from the N2 generation only; however, once the anti-mitotic effect of the HCV transgene was bridged in tumors, then mitosis was enhanced in both female and male mice from the N2 generation (Fig. 3A and Fig. 3C). This effect was more prominent in female mice, although it did not reach statistical significance. The presence of the highly proliferative tumors with diameters of up to 1 cm in Mdr2-KO/HCV-Tg females indicates a possible reason for the appearance of large tumors with diameters over 1 cm in this experimental group (Fig. 1C) as well. In the N7 females, the anti-mitotic effect of the HCV transgene on non-tumorous liver tissue was absent, and the mitotic activities of tumors in the Mdr2-KO and Mdr2-KO/HCV-Tg females were similar to each other and to those of the Mdr2-KO females from the N2 generation (Fig. 3A). The levels of apoptosis in tumors were similar between N2 and N7 females, independent of the presence of the HCV transgene (Fig. 3B).

### Gene expression profiling analysis

**Figure 4:**
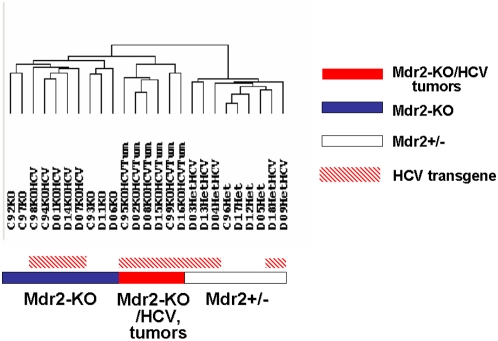
Hierarchical clustering of expression profiles of liver tissue samples subjected to whole genome expression profiling. Average linkage hierarchical clustering of liver tissue samples from females of the N2 generation was performed on 5,000 genes with highest STD values using the software from Michael Eisen's Laboratory (Lawrence Berkeley National Laboratory and University of California, Berkeley; http://rana.lbl.gov/EisenSoftware.htm). The Mdr2 genotype and the tumor phenotype were major clustering forces, whereas the contribution of the HCV transgene in clustering was only marginal.

In an effort to delineate the molecular mechanisms associated with the tumor promoting effect of the HCV transgene in Mdr2-KO/HCV-Tg N2 females, we performed a genome scale gene expression profiling on livers of 14-month-old female mice. Non-tumorous liver tissues of Mdr2-KO, Mdr2^+/−^, Mdr2^+/−^/HCV-Tg, and Mdr2-KO/HCV-Tg female mice from the N2 generation, as well as all large tumors (diameters over 1 cm) of Mdr2-KO/HCV-Tg females, were analyzed (Fig. 4). In hierarchical clustering, the Mdr2 genotype and the tumor phenotype were major clustering forces separating the samples into three main clusters: Mdr2-KO, Mdr2^+/−^ and tumors. Remarkably, in the cluster of healthy Mdr2^+/−^ mice, the HCV transgene was a destabilizing factor, whereas in the chronically inflamed Mdr2-KO background, it was a stabilizing factor providing clusterization of all HCV-Tg samples into one subcluster. Similarly, Mdr2-KO mutation was the main factor contributing to the alternative splicing between the experimental groups (Table S1). Overall, the contribution of the HCV transgene in both differential gene expression (Fig. S2) and alternative splicing (Table S1) was only marginal. However, supervised analysis of genes with a tendency to differential expression between Mdr2-KO and Mdr2-KO/HCV-Tg samples revealed a substantial overlap of pathways affected by the HCV transgene and the Mdr2-KO mutation (Table S2). This overlap is comprised of all arms of the immune system, including the innate, humoral and, to some extent, the adaptive responses; many of these genes were also similarly differentially expressed between Mdr2^+/−^ and Mdr2^+/−^/HCV-Tg samples (data not shown).

### Confirmation of differential gene expression

Differential expression of selected genes was further confirmed by RT-PCR (Table 3). These genes are involved in the regulation of immune/inflammatory response (Lgals1, Ptgds, Tff2, Ubd), tumor angiogenesis (Lgals1, Esm1, Ndrg1), epidermal growth factor signaling (Erbb4, Nrg1), response to tissue injury (Tff2, Vnn1), and gene transcription (Id3, Ifrd1). Some of these genes (Lgals1, Nrg1, Ubd) have been already identified previously as HCV-dependent [22]–[25]. More detailed information on the confirmed genes and their potential roles in carcinogenesis can be found in Table 3.

**Table 3:** Confirmation of the differential gene expression by RT-PCR.

Gene symbol	Accession number	Differential expression$	Ttest&	Gene name	Known or suggested role of the gene in cancer
Cyp4a31@	NM_201640	KO>NT	p = 0.001	Cytochrome P450, family 4, subfamily a, polypeptide 31	Iron ion binding [49].
Erbb4@	NM_010154	T>NT	p = 0.003	V-erb-a erythroblastic leukemia viral oncogene homolog 4	Activated by neuregulins; increases proliferation potential of cancer cells [50].
Esm1@	NM_023612	T>NT	p = 0.009	Endothelial cell-specific molecule 1	Preferentially expressed in tumor endothelium [51].
Gnmt#	NM_010321	T<NT		Glycine N-methyltransferase	Downregulated in HCC [52].
Id3@	NM_008321	T<NT	p = 0.04	Inhibitor of DNA binding 3	Expression in HCC decreases with tumor dedifferentiation [53].
Ifrd1@	NM_013562	T>NT	p = 0.04	Interferon-related developmental regulator 1	Regulates differentiation of epithelial cells [54].
Ndrg1@	NM_008681	T>NT	p = 0.02	N-myc down-stream regu-lated gene 1	Mediates prolife-ration and invasion of HCC cells [55].
Nrg1@	NM_178591	T>NT	p = 0.006	Neuregulin 1	Upregulated in HCV-induced HCC [24].
Ptgds@	NM_008963	KO<NT	p = 0.04	Prostaglandin D2 synthase	Antiproliferative in prostate cancer [56].
Sox4@ #	NM_009238	KO>NT	p = 0.003	SRY-box containing gene 4	Positive regulator of HCC cells' apoptosis [57].
Styk1@	NM_172891	T>NT	p = 0.006	Serine/threonine/tyrosine kinase 1	Induces tumorige-nesis and metastasis in nude mice [58].
Tff2@	NM_009363	KO<NT	p = 0.02	Trefoil factor 2	Promotes epithelial-cell restitution, cont-rols inflammation and systemic immune responses [59].
Ubd@	NM_023137	T>NT	p = 0.03	Ubiquitin D (FAT10)	Upregulated in various cancers including HCC [27], resulting in chromo-some instability [60].
Vnn1@	NM_011704	T>NT	p = 0.01	vanin 1	Regulates response to oxidative injury in epithelial cells [61].

@ Confirmed by semi-quantitative RT-PCR.

# Confirmed by real time RT-PCR (Sox4 was confirmed by both methods).

$ T – tumorous and NT – matched non-tumorous liver tissues of Mdr2-KO/HCV-Tg females; KO - non-tumorous liver tissues of Mdr2-KO females.

& p-values for semi-quantitative RT-PCR are provided (see “Materials and methods”).

**Figure 5:**
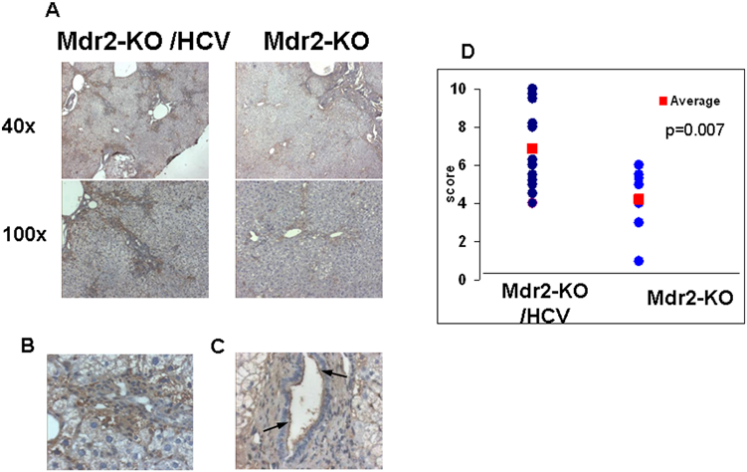
Higher expression of galectin-1 in the livers of Mdr2-KO/HCV-Tg N2 females. (A–C) Immunohistochemical staining of non-tumorous liver tissues of 14-month-old Mdr2-KO and Mdr2-KO/HCV-Tg females from N2 generation with an antibody specific to galectin-1. (A) Left panel: Mdr2-KO/HCV-Tg; right panel: Mdr2-KO females. Magnification: upper panel ×40, lower panel ×100. (B) Cytoplasmic staining of hepatocytes in Mdr2-KO/HCV livers. Magnification ×400. (C) Staining of luminal side of cholangiocytes' membranes (indicated by arrows) in Mdr2-KO/HCV livers. Magnification ×400. (D) Scores of the immunostaining level for all liver samples in each group are shown in blue circles; average scores of each group are shown in red squares; p = 0.007 between the groups.

**Figure 6:**
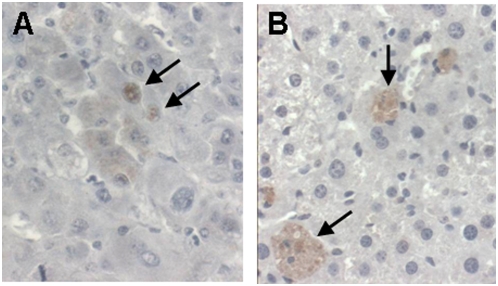
Nuclear expression of diubiquitin in tumors and its cytoplasmic expression in non-tumorous hepatocytes. Immunohistochemical staining of 14-months-old Mdr2-KO/HCV-Tg N2 females with an antibody specific to diubiquitin. (A) Nuclear staining of hepatocytes in tumorous tissue (arrows). (B) Cytoplasmic staining of hepatocytes in non-tumorous liver tissue (arrows). Magnification: ×400.

Up-regulation of two proteins, galectin-1 (encoded by the Lgals1 gene) and diubiquitin (encoded by the Ubd/Fat10 gene), was confirmed by immunohistochemistry. Galectin-1 modulates innate and adaptive immune responses; its over-expression in many types of tumors and/or surrounding tissues promotes tumor progression by inhibition of anti-tumor immune response [26]. Galectin-1 was overexpressed in non-tumorous liver tissue of Mdr2-KO/HCV-Tg mice, mostly in the cytoplasm of hepatocytes in zones 1 and 3, in endothelial cells, and on the luminal side of cholangiocyte membranes (Fig. 5). Diubiquitin is involved in the maintenance of spindle integrity during mitosis and is highly upregulated in human HCC [27]; it was detected mostly in the cytoplasm of hepatocytes in the tumors of Mdr2-KO/HCV-Tg mice, and, to a lesser degree, in non-tumorous liver tissues of Mdr2-KO mice with or without the HCV transgene (Fig. 6). Remarkably, diubiquitin was detected in the hepatocyte nuclei only in large tumors of Mdr2-KO/HCV-Tg mice, similar to its nuclear localization in human HCC [27].

### Testing of other known effects of the HCV transgene expression

**Figure 7:**
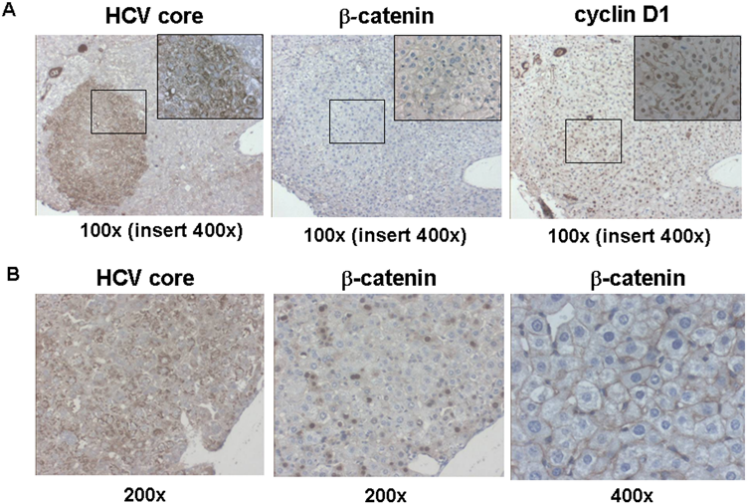
Immunohistochemical detection of the HCV core, β-catenin and cyclin D1 proteins in the liver of Mdr2-KO/HCV-Tg mice. (A) A typical example of the absence of correlation between the expression of the HCV core and either nuclear level of cyclin D1, or cytoplasmic/nuclear level of β-catenin proteins in hepatocytes. Magnification: ×100. Inserts contain the framed regions magnified ×400. (B) A rare example of the correlation between the expression of the HCV core and nuclear level of β-catenin proteins in hepatocytes (first two panels; magnification: ×200). Third panel: a typical membrane-associated pattern of β-catenin expression in hepatocytes of Mdr2-KO/HCV-Tg mice. Magnification: ×400.

We searched for a correlation between the local level of the HCV transgene expression (revealed by immunohistochemical detection of the HCV core protein) and other oncogenic markers. There was no correlation between the level of HCV transgene expression and the presence of dysplastic nodules in Mdr2-KO/HCV-Tg mice. It was shown previously that HCV-associated HCC is characterized by increased frequency of β-catenin mutations and by activation of β-catenin signaling [28], [29]. We reported recently that in the non-tumorous liver of Mdr2-KO mice, β-catenin signaling is inhibited while the nuclear level of cyclin D1 is increased [18]. The expression level of the HCV transgene in hepatocytes of Mdr2-KO/HCV-Tg mice correlated neither with the nuclear level of cyclin D1 nor with the cytoplasmic/nuclear level of β-catenin (a typical example shown in Fig. 7A). However, in some rare cases, high HCV transgene expression correlated with nuclear localization of β-catenin (Fig. 7B). One of the six tumors analyzed by whole genome gene expression profiling was characterized by a prominent upregulation of the β-catenin-activated genes and by rare scattered β-catenin-positive hepatocyte nuclei (data not shown).

## Discussion

The molecular mechanisms of HCV-induced HCC development are still not well understood; one of the main questions is whether HCC develops due to chronic inflammation or whether HCV proteins are endowed with some oncogenic activity. Multiple cell culture models have demonstrated the oncogenic potential of several HCV proteins; however, most transgenic mouse models expressing HCV proteins did not develop HCC [2]. Similarly, none of the two previously generated transgenic mouse models expressing the whole HCV polyprotein is developing HCC [7], [9], [10]. Taking into account that in mouse transgenic models there is no host immune reaction against expressed viral proteins, we explored whether HCV proteins would accelerate inflammation-associated hepatocarcinogenesis. We bred mice expressing the whole HCV polyprotein with Mdr2-KO mice, a model of inflammation-associated HCC, and monitored HCC development in two generations of hybrid mice: N2 and N7, containing 25% and 0.8% of C57Bl/6 genetic background, respectively. We demonstrated, for the first time, that a transgene expressing the whole HCV polyprotein accelerates inflammation-associated hepatocarcinogenesis *in vivo* in a host genetic background-dependent manner: only in the N2 generation and only in females. Remarkably, our previous studies of Mdr2-KO mice [15] demonstrated that chronic liver disease at an early precancerous stage in this HCC model shares many features characteristic of chronic HCV infection and HCV-induced cirrhosis in patients: hepatocyte G1 arrest and up-regulation of genes Mcm2 [30], Cxcl9, Lgals3, Iqgap1, Lum, Col6a3, Igfbp7, Vim, Ablim, Mpp1 [31], Igfbp1, Itgb1, Jun, Ctgf [32]. This similarity was further confirmed by a significant overlap of pathways affected by the HCV transgene and the Mdr2-KO mutation revealed in the current study (Table S2).

The 25% of C57Bl/6 genetic background had a tumor-suppressive effect on HCC development in the N2 mice not having the HCV transgene, in accord with a well known low susceptibility of the C57Bl/6 mice to tumors [33]. Thus, in the PyMT mouse model of breast cancer, C57Bl/6 mice and F1 hybrids between the C57Bl/6 and FVB/N strains had more prolonged primary tumor latency than the FVB/N mice [34], [35]. Remarkably, the effect of inducible nitric oxide synthase deficiency on mammary tumor development and metastasis was prominent in the C57Bl/6 genetic background, where tumors developed slowly, but was not observed in the FVB/N genetic background, where tumors developed quickly [34]. In the Ras-induced skin squamous carcinoma model, the low susceptibility of the C57Bl/6 strain and the high susceptibility of the FVB/N had been attributed to a polymorphism in the Ptch gene [36]. Interestingly, it was recently demonstrated that inactivation of the Nox2 gene in the mutant Sod1 mouse model for amyotrophic lateral sclerosis significantly increased survival when mice were of mixed genetic background [37], but had very modest effect on the inbred C57Bl/6 strain [38]. In accord with these findings, our data demonstrate that for mouse models of human diseases, a specific genetic background may be found where manifestations of the disease and/or therapeutic treatments will be the most prominent.

The female-specific effect of the HCV transgene raises a question about the relevance of this model to human disease, since it is widely accepted that human HCC is more prevalent in males. The typical average ratio of males∶females among HCC patients is 3∶1 [39], however, this proportion depends on the etiology of the disease: among HBV-infected patients, it is about 6∶1, whereas among HCV-infected patients, it is about 1.7∶1 [40]. Moreover, when other higher HCC risk factors in males such as alcohol consumption are taken into account, the normalized risk of HCC among HCV-infected patients may be higher for females [41]. Therefore, the appearance of the tumor-promoting effect of the HCV transgene seen only in females in the described model may have a physiological significance. Recently, it was demonstrated that in chemically induced hepatocarcinogenesis in C57Bl/6 mice, the estrogen-mediated inhibition of IL-6 production is responsible for a lower risk of liver cancer in females [3]. However, in the Mdr2-KO mice, the risk for liver cancer is similar in both genders [11], [42], suggesting that there are probably other mechanisms regulating hepatocarcinogenesis in this model of chronic inflammation-associated HCC.

Comparison of mitotic and apoptotic events in non-tumorous liver tissues of the Mdr2-KO and Mdr2-KO/HCV-Tg mice revealed a statistically significant decrease of mitosis and apoptosis in the Mdr2-KO/HCV-Tg females from the N2 generation. Interestingly, similar effects of the transgene expressing the HCV open reading frame on the untransformed human hepatocytes have been demonstrated recently: absence of the direct oncogenic activity, inhibition of cell proliferation, and up-regulation of genes involved in innate immune response/inflammation [43]. However, in tumors, an inverse tendency took place: Mdr2-KO/HCV-Tg mice had higher levels of both mitosis and apoptosis, whereas in Mdr2-KO mice, the levels of mitosis and apoptosis in non-tumorous and tumorous tissues were similar. These findings suggest that the accelerating effect of the HCV transgene on hepatocarcinogenesis in this model is most probably due to promotion of tumor proliferation by the HCV transgene. Similarly, the difference in liver cancer susceptibility between the C3H/He and C57Bl/6 strains has been attributed to a dissimilar tumor cell proliferation between these two groups, but not to intensity of apoptosis in their livers [44]. High standard errors observed in the current study for many parameters at the tumorous stage of the disease (e.g., hepatocyte's apoptotic and mitotic frequencies) were most probably caused by a prolonged chronic liver disease associated with induction of different homeostatic mechanisms in the mutant mice.

The tumor promoting effect of the HCV transgene may be explained both by upregulation of pro-tumorigenic genes as Ubd, Erbb4, Nrg1, Ndrg1, Styk1 (Table 3), and by evasion of host defense response by viral proteins [45], [46]. Over-expression of the galectin-1 protein in the liver of Mdr2-KO/HCV-Tg mice may also cause inhibition of anti-tumor immune response [26]. One of the known differences between the C57Bl/6 and FVB/N inbred strains is that the latter is C5 complement-deficient. The important role of C5 and its receptor C5a in host inflammatory reactions [47] suggests that this factor may be responsible for the observed differences in HCC development between the N2 and N7 generations. Interestingly, comparison of differentially expressed genes (Mdr2-KO relative to control mice) of the N2 generation and of FVB/N Mdr2-KO mice aged 12 months [15] or 16 months [18], previously published by our group, revealed significant up-regulation of the complement component C9 in the Mdr2-KO livers of the N2 generation (4-fold; p = 0.0066), but not in FVB/N Mdr2-KO mice. And conversely, inflammatory markers Aif1, Cd36, Cd44 and Spp1 (osteopontin) were up-regulated in FVB/N Mdr2-KO mice at all tested ages, but not in the Mdr2-KO livers of the N2 generation.

This study demonstrates that the HCV transgene accelerated inflammation-associated hepatocarcinogenesis *in vivo* in a host genetic background-dependent manner, thus emphasizing the significance of the host genotype in HCV manifestations. This accelerating effect was prominent in the mixed, partially tumor-suppressive, FVB/N×C57Bl/6 genetic background, and is most probably due to promotion of tumor proliferation by the HCV transgene. Despite multiple reports on the direct oncogenic activities of different HCV proteins *in vitro*, in mouse models *in vivo*, these data are contradictory, whereas data on the tumor promoting effect of HCV proteins are much more consistent [10], [23], [48]. Our data suggest that the selected Mdr2-KO mouse model was appropriate for this specific investigation aimed to determine which of the potential different factors contributed mainly to HCC development with special emphasis on the inflammatory process in the Mdr2-KO mice lacking the HCV transgene as its main contribution to the model. The role of specific factors contributing to tumor promoting activity of the HCV transgene: sex, known genes that differ between the two studied mouse strains, and differentially expressed regulatory genes revealed by our gene expression profiling analysis requires further investigation.

## Supporting Information

Figure S1 Scheme of the breeding of B6/HCV and Mdr2-KO mice to generate Mdr2-KO/HCV-Tg mice.
(1.53 MB TIF)

Figure S2 The effect of Mdr2-KO, HCV-Tg and tumor phenotype on differential gene expression between experimental groups. The result of the SPIN analysis performed on 1,000 genes with the highest standard deviation. The left panel displays the color-coded standardized Euclidean distance matrix of samples and the right panel - the expression matrix of genes. Similar to hierarchical clustering (Figure 4), clustering was determined mainly by the Mdr2 genotype and the tumor phenotype, whereas the contribution of the HCV transgene was only marginal.
(3.16 MB TIF)

Table S1 The effect of Mdr2-KO, HCV-Tg, and tumor phenotype on alternative splicing. Numbers before slash represent the amount of probe-sets with 2-fold or 1.8-fold change in the relative level of exon inclusion, determined as statistically significant by either paired t-test (for tumors versus non-tumorous tissues), or two-way ANOVA (for all other categories). Numbers after slash represent the amount of unique genes represented by the selected probe-sets. * The data for alternative splicing in tumors could not be directly compared with other categories due to the use of a different method of calculation.
(0.03 MB DOC)

Table S2 Substantial overlapping of pathways affected by the HCV transgene and by the Mdr2-KO mutation. The SPIN analysis of the complete set of samples performed in the space of the 1,000 most variable genes resulted in separation of the samples into 3 groups: non-tumorous Mdr2-KO, Mdr2(+/−), and tumorous Mdr2-KO/HCV-Tg samples (Fig. S2, left). The non-tumorous Mdr2-KO set was roughly separated into Mdr2-KO/HCV-Tg and Mdr2-KO subsets. The SPIN analysis of the selected gene-set in the space of all Mdr2-KO samples enabled isolation of genes with tendency to differential expression between HCV-Tg and non-HCV samples. The lists of genes with tendency to up- and down-regulation in Mdr2-KO/HCV-Tg samples were submitted to functional analysis by GO categories using the DAVID tool. These results were compared to the results of functional analysis by GO categories of genes differentially expressed between Mdr2-KO and Mdr2(+/−) non-HCV samples (threshold 1.85). For each enriched GO term, the number of related genes, their percent in the analyzed gene list and the significance of the enrichment are shown.
(0.03 MB DOC)
