# Supplementary figures and images for: HCV Tumor Promoting Effect Is Dependent on Host Genetic Background
Source: PLoS One. 2009 Apr 2;4(4):e5025. doi: 10.1371/journal.pone.0005025 (PMC2660413; doi:10.1371/journal.pone.0005025)

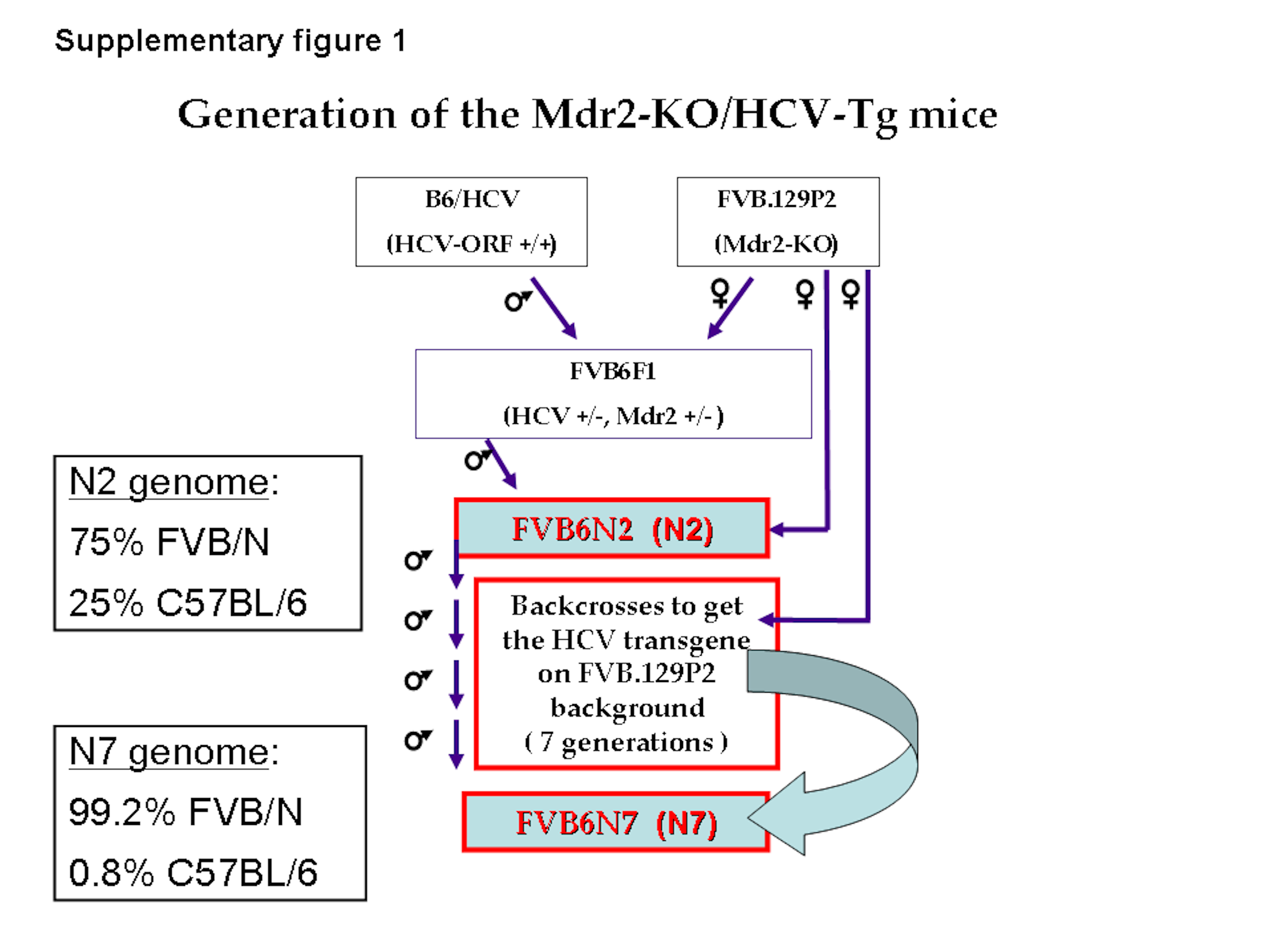

Supplement: Figure S1 — Scheme of the breeding of B6/HCV and Mdr2-KO mice to generate Mdr2-KO/HCV-Tg mice. (1.53 MB TIF) [file pone.0005025.s001.tif]

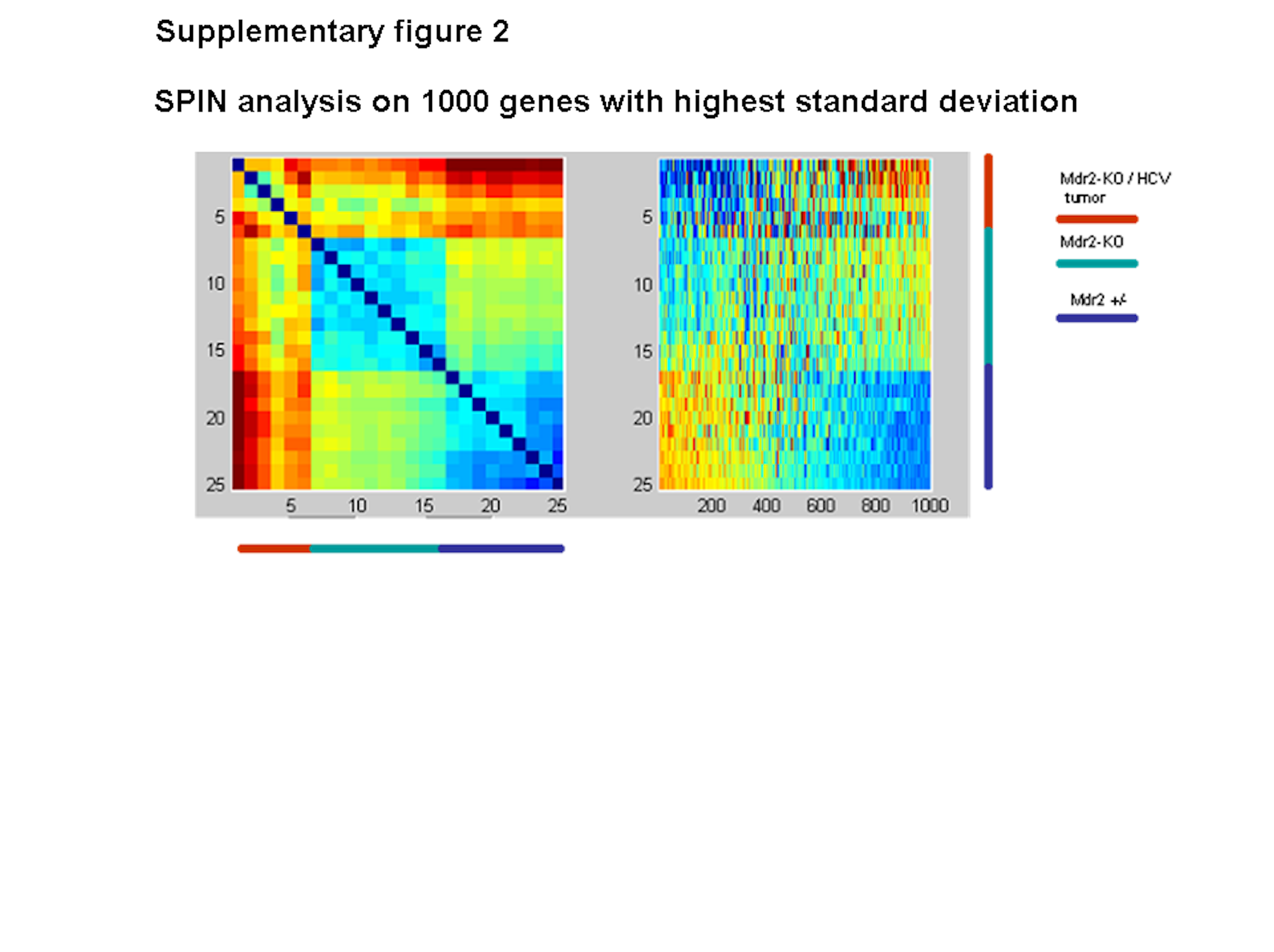

Supplement: Figure S2 — The effect of Mdr2-KO, HCV-Tg and tumor phenotype on differential gene expression between experimental groups. The result of the SPIN analysis performed on 1,000 genes with the highest standard deviation. The left panel displays the color-coded standardized Euclidean distance matrix of samples and the right panel - the expression matrix of genes. Similar to hierarchical clustering (Figure 4), clustering was determined mainly by the Mdr2 genotype and the tumor phenotype, whereas the contribution of the HCV transgene was only marginal. (3.16 MB TIF) [file pone.0005025.s002.tif]
