# Supplementary material for: HCV Tumor Promoting Effect Is Dependent on Host Genetic Background
Source: PLoS One. 2009 Apr 2;4(4):e5025. doi: 10.1371/journal.pone.0005025 (PMC2660413; doi:10.1371/journal.pone.0005025)
Supplement: Table S1 — The effect of Mdr2-KO, HCV-Tg, and tumor phenotype on alternative splicing. Numbers before slash represent the amount of probe-sets with 2-fold or 1.8-fold change in the relative level of exon inclusion, determined as statistically significant by either paired t-test (for tumors versus non-tumorous tissues), or two-way ANOVA (for all other categories). Numbers after slash represent the amount of unique genes represented by the selected probe-sets. * The data for alternative splicing in tumors could not be directly compared with other categories due to the use of a different method of calculation. (0.03 MB DOC) [file pone.0005025.s003.doc]

**Supplementary Table 1.**

| **Mdr2 and HCV cooperative effect in Mdr2(+/-)** | **Mdr2 and HCV cooperative effect in Mdr2-KO** | **Mdr2 effect** | **HCV effect** | **Tumors versus**  **non-tumorous tissues** * |  |
| --- | --- | --- | --- | --- | --- |
| 22 / 21 | 29 / 24 | 2088 / 490 | 31 / 24 | 535 / 240 | **2-fold** |
| 67 / 63 | 77 / 67 | 3614 / 845 | 97 / 75 | 990 / 485 | **1.8-fold** |

The effect of Mdr2-KO, HCV-Tg, and tumor phenotype on alternative splicing. Numbers before slash represent the amount of probe-sets with 2-fold or 1.8-fold change in the relative level of exon inclusion, determined as statistically significant by either paired t-test (for tumors versus non-tumorous tissues), or two-way ANOVA (for all other categories). Numbers after slash represent the amount of unique genes represented by the selected probe-sets. ***** The data for alternative splicing in tumors could not be directly compared with other categories due to the use of a different method of calculation.
