# Supplementary material for: HCV Tumor Promoting Effect Is Dependent on Host Genetic Background
Source: PLoS One. 2009 Apr 2;4(4):e5025. doi: 10.1371/journal.pone.0005025 (PMC2660413; doi:10.1371/journal.pone.0005025)
Supplement: Table S2 — Substantial overlapping of pathways affected by the HCV transgene and by the Mdr2-KO mutation. The SPIN analysis of the complete set of samples performed in the space of the 1,000 most variable genes resulted in separation of the samples into 3 groups: non-tumorous Mdr2-KO, Mdr2(+/−), and tumorous Mdr2-KO/HCV-Tg samples (Fig. S2, left). The non-tumorous Mdr2-KO set was roughly separated into Mdr2-KO/HCV-Tg and Mdr2-KO subsets. The SPIN analysis of the selected gene-set in the space of all Mdr2-KO samples enabled isolation of genes with tendency to differential expression between HCV-Tg and non-HCV samples. The lists of genes with tendency to up- and down-regulation in Mdr2-KO/HCV-Tg samples were submitted to functional analysis by GO categories using the DAVID tool. These results were compared to the results of functional analysis by GO categories of genes differentially expressed between Mdr2-KO and Mdr2(+/−) non-HCV samples (threshold 1.85). For each enriched GO term, the number of related genes, their percent in the analyzed gene list and the significance of the enrichment are shown. (0.03 MB DOC) [file pone.0005025.s004.doc]

**Supplementary Table 2.**

Substantial overlapping of pathways affected by the HCV transgene and by the Mdr2-KO mutation. The SPIN analysis of the complete set of samples performed in the space of the 1,000 most variable genes resulted in separation of the samples into 3 groups: non-tumorous Mdr2-KO, Mdr2(+/-), and tumorous Mdr2-KO/HCV-Tg samples (Supplementary Fig. 2, left). The non-tumorous Mdr2-KO set was roughly separated into Mdr2-KO/HCV-Tg and Mdr2-KO subsets. The SPIN analysis of the selected gene-set in the space of all Mdr2-KO samples enabled isolation of genes with tendency to differential expression between HCV-Tg and non-HCV samples. The lists of genes with tendency to up- and down-regulation in Mdr2-KO/HCV-Tg samples were submitted to functional analysis by GO categories using the DAVID tool. These results were compared to the results of functional analysis by GO categories of genes differentially expressed between Mdr2-KO and Mdr2(+/-) non-HCV samples (threshold 1.85). For each enriched GO term, the number of related genes, their percent in the analyzed gene list and the significance of the enrichment are shown.
